# Pre-clinical evaluation of a *P. berghei*-based whole-sporozoite malaria vaccine candidate

**DOI:** 10.1038/s41541-018-0091-3

**Published:** 2018-11-27

**Authors:** António M. Mendes, Isaie J. Reuling, Carolina M. Andrade, Thomas D. Otto, Marta Machado, Filipa Teixeira, Joana Pissarra, Nataniel Gonçalves-Rosa, Dolores Bonaparte, João Sinfrónio, Mandy Sanders, Chris J. Janse, Shahid M. Khan, Chris I. Newbold, Matthew Berriman, Cynthia K. Lee, Yimin Wu, Christian F. Ockenhouse, Robert W. Sauerwein, Miguel Prudêncio

**Affiliations:** 1 Instituto de Medicina Molecular, Faculdade de Medicina, Universidade de Lisboa, Avenida Professor Egas Moniz, 1649-028 Lisboa, Portugal; 2 Department of Medical Microbiology, Radboud University Medical Center, Geert Grooteplein 28, Microbiology 268, 6500 HB Nijmegen, The Netherlands; 3 Parasite Genomics, Wellcome Trust Sanger Institute, Hinxton, Cambridge, CB10 1SA UK; 4 Leiden Malaria Research Group, Parasitology, Center of Infectious Diseases, Leiden University Medical Center, Leiden, The Netherlands; 5 PATH’s Malaria Vaccine Initiative, 455 Massachusetts Ave, Washington, DC 20001 USA; 6 Present Address: Centre of Immunobiology, Institute of Infection, Immunity & Inflammation, MVLS, University of Glasgow, Glasgow, UK

## Abstract

Whole-sporozoite vaccination/immunization induces high levels of protective immunity in both rodent models of malaria and in humans. Recently, we generated a transgenic line of the rodent malaria parasite *P. berghei* (*Pb*) that expresses the *P. falciparum* (*Pf*) circumsporozoite protein (*Pf*CS), and showed that this parasite line (*Pb*Vac) was capable of (1) infecting and developing in human hepatocytes but not in human erythrocytes, and (2) inducing neutralizing antibodies against the human *Pf* parasite. Here, we analyzed *Pb*Vac in detail and developed tools necessary for its use in clinical studies. A microbiological contaminant-free Master Cell Bank of *Pb*Vac parasites was generated through a process of cyclic propagation and clonal expansion in mice and mosquitoes and was genetically characterized. A highly sensitive qRT-PCR-based method was established that enables *Pb*Vac parasite detection and quantification at low parasite densities in vivo. This method was employed in a biodistribution study in a rabbit model, revealing that the parasite is only present at the site of administration and in the liver up to 48 h post infection and is no longer detectable at any site 10 days after administration. An extensive toxicology investigation carried out in rabbits further showed the absence of *Pb*Vac-related toxicity. In vivo drug sensitivity assays employing rodent models of infection showed that both the liver and the blood stage forms of *Pb*Vac were completely eliminated by Malarone^®^ treatment. Collectively, our pre-clinical safety assessment demonstrates that *Pb*Vac possesses all characteristics necessary to advance into clinical evaluation.

## Malaria: Pre-clinical characterization of transgenic malaria vaccine

*Pb*Vac is a transgenic malaria parasite expressing circumsporozoite antigen from the human parasite *Plasmodium falciparum*. *Pb*Vac elicits neutralizing *P. falciparum* antibodies and can infect human hepatocytes but not erythrocytes, suggesting that humans would be non-permissive. Miguel Prudêncio and colleagues at the Institute of Molecular Medicine in Lisbon perform a detailed *in vivo* analysis and toxicology of *Pb*Vac. Extensive biodistribution analysis using a highly sensitive qPCR in non-permissive rabbit hosts shows *Pb*Vac are present at the initial bite site early on with later appearance in the liver, but by day 10 is undetectable. Importantly no *Pb*Vac could be detected in the blood at any time-point. *Pb*Vac was well tolerated with no apparent pathological signatures. In permissive mouse hosts *Pb*Vac could be effectively eliminated from both the blood and liver and could thereby act as a potential clinical ‘safety net’ in the event of an erythrocytic stage or persistence in the liver.

## Introduction

Despite a recent decrease in malaria-related mortality, the goal of eradication remains distant and is unlikely to be achieved in the absence of an effective vaccine against this disease. Whole-sporozoite (WSp) vaccination approaches have been shown to elicit sterile protection against malaria both in rodent models and in humans. Protection mediated by such vaccine candidates relies on the immune responses elicited by the parasite’s pre-erythrocytic forms, following administration of live sporozoites to the mammalian host. WSp-based malaria immunization generally employs *P. falciparum* (*Pf*) sporozoites rendered safe by radiation or genetic attenuation, or by concomitant prophylactic administration of antimalarial drugs acting on the erythrocytic stage of the parasite^1^.

The first draft genome of *Pb* ANKA (*Pb*A) was published in 2005,^2^ followed by further sequencing, re-assembly and annotation of what has since constituted the reference *Pb*A genomic sequence.^3^ Comparisons of the *Pb*A genome sequence with that of the canonical reference *Pf* clone 3D7^4^ revealed that a high percentage of the proteins predicted for the former parasite have orthologs in the latter, as well as the near absence of polymorphisms within the genomes of *Pb* isolates.^5^ The relative similarity between the genomes of *Pb* and human-infective *Plasmodium* spp., *Pb*’s non-pathogenicity to humans, the ability of *Pb* sporozoites to infect various types of hepatic cells and cell lines, the availability of rodent models to study *Pb* infection in vivo, and *Pb*’s amenability to genetic modification, have made this parasite one of the preferred models for the investigation of *Plasmodium* infection and the analysis of *Plasmodium* gene function.

Among the many achievements that *Pb* parasites have enabled, they have been instrumental in the development of current WSp malaria vaccine candidates. In fact, protection conferred by radiation attenuated sporozoites (RAS), which are unable to develop inside liver cells, was initially demonstrated in mice using irradiated wild-type *Pb* parasites,^6^ paving the way for the development of *Pf* RAS vaccine candidates.^7–9^ Sporozoites can also be attenuated by deletion of specific genes, leading to the parasite’s developmental arrest in the liver,^10^ an immunization strategy that is awaiting clinical validation.^11,12^ Typically, although not always,^13^ genes targeted for deletion in *Pf* to generate genetically attenuated parasites (GAP) are homologs of genes primarily identified in rodent parasites as critical for their hepatic development.^14^ Finally, the administration of *Pf* sporozoites under a chemoprophylactic regimen of an antimalarial drug (CPS), which kills the parasite’s blood forms, and has also been shown to confer long lasting protection in humans,^15,16^ was equally based on previous studies in rodents, employing *Pb* sporozoites administered concomitantly with a prophylactic dose of chloroquine.^17^

Collectively, the studies outlined above illustrate the pivotal role played by wild-type and genetically modified *Pb* in the field of WSp malaria vaccination. However, a significant advance occurred recently, with a genetically modified *Pb* parasite, termed *Pb*Vac, proposed as a WSp vaccine against human malaria in and of itself, rather than as a surrogate for *Pf*-based vaccination or a tool to assess immune responses elicited by *Pf* vaccines. *Pb*Vac has been engineered to express the immunodominant *Pf* antigen, the circumsporozoite protein (*Pf*CS), flanked by the *Pb* pre-erythrocytic stage-specific promoter, UIS4 (upregulated in infective sporozoites 4).^10^ *Pb*Vac infects and develops in human hepatocytes but not in human red blood cells, and is capable of eliciting both *Pb*/*Pf* cross-species and *Pf*CS-dependent immune responses against the human-infective *Pf* parasite.^18^

The results outlined above warrant the clinical evaluation of *Pb*Vac. However, such an endeavor demands an encompassing characterization of the *Pb*Vac parasite as well as the development and/or adaptation of methodologies ensuring its safety for human use. Here, we describe the establishment of a microbiological contaminant free and genetically stable *Pb*Vac parasite Master Cell Bank (MCB), as well as a highly sensitive qRT-PCR based methodology for detection and quantification of *Pb*Vac parasites in tissues and organs. Parasites from this MCB were used in an extensive array of biodistribution, toxicology, and drug sensitivity assays that ascertain its amenability to clinical use. Collectively, these results paved a way for the evaluation of *Pb*Vac in Phase I/IIa clinical trials aimed at assessing its safety, tolerability, and protective efficacy in humans.

## Results

### Generation and analyses of a PbVac master cell bank

**Fig. 1:**
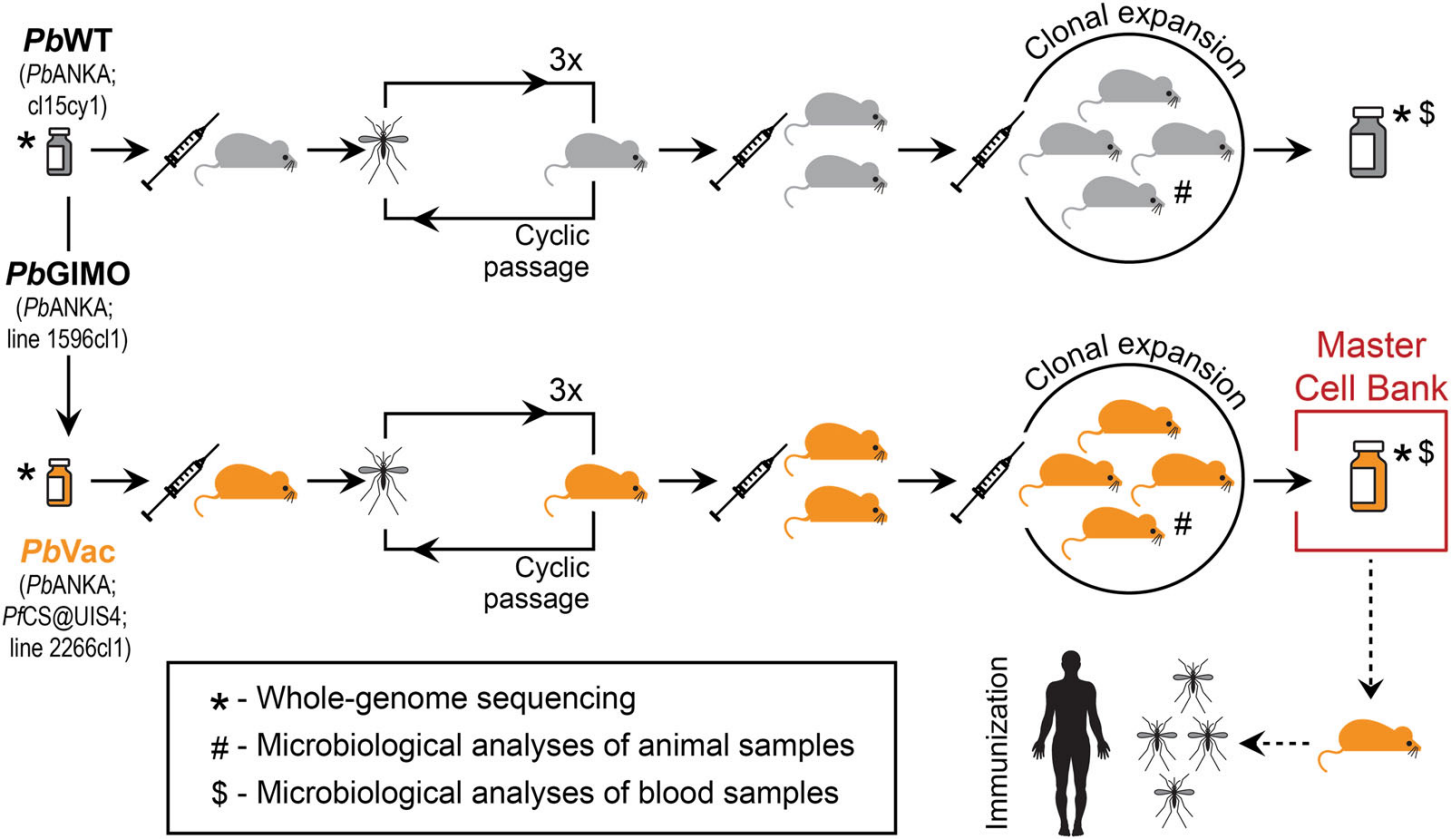
Construction of *Pb*Vac MCB. *Pb*Vac parasites underwent 3 consecutive cyclic passages through mice and mosquitoes prior to clonal expansion in a final batch of mice. Blood collected from these mice was aliquoted and stored to constitute the *Pb*Vac MCB, and tested for microbiological contamination. In parallel, the parental wild-type *Pb* parasite underwent a similar procedure and was subsequently aliquoted and stored. The whole genomes of both parasites were sequenced before and after cyclical propagation and clonal expansion. SPF-certified BALB/cByJ mice were used in all steps of the procedure

**Fig. 2:**
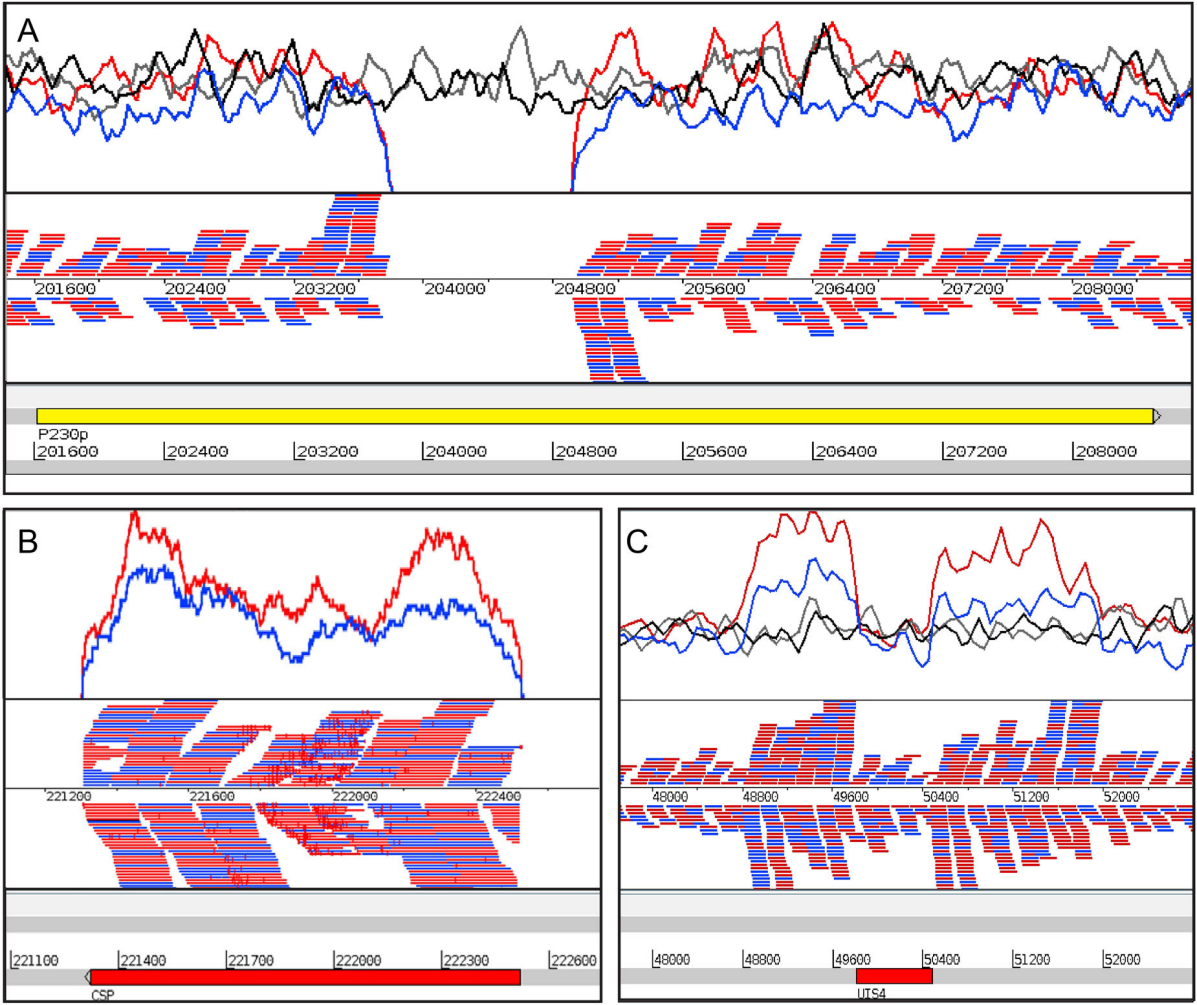
Whole-genome sequencing of wild-type *Pb* and *Pb*Vac parasites before and after cyclical propagation and clonal expansion. **a** Insertion site of the construct in the P230p locus of *Pb*’s chromosome 3. **b** The *Pf*CS gene is covered by reads from the *Pb*Vac parasite lines, showing that it is contained in the genome of the genetically modified parasite. **c** Artemis screenshot showing the copy number variation of the *Pb*UIS4 gene in the wild-type *Pb* and *Pb*Vac parasites (top) and the “not properly paired” reads of the transgenic parasite lines (bottom). For each panel, the coverage plot is represented at the top and the mapped reads are shown at the bottom. Black: wild-type *Pb* prior to cyclic propagation; gray: wild-type *Pb* after cyclic propagation and clonal expansion; blue: *Pb*Vac prior to cyclic propagation; red: *Pb*Vac after cyclic propagation and clonal expansion

MCB were constructed for the parental wild-type *Pb* and the cloned *Pb*Vac parasites, as described in the Methods section (Fig. 1). Analyses of fecal pellets, oral swabs, and fur swabs of SPF Balb/c mice employed throughout the study confirmed the absence of microbiological contamination (Table S1). MCB samples were analyzed and found to be free of adventitious virus, as well as mouse- and human-infective microbiological contaminants (Table S2). The entire genomes of the *Pb* and *Pb*Vac parasites collected prior to and following cyclical propagation were subsequently sequenced, in order to extensively characterize the genetic differences between the wild-type and the genetically modified *Pb* parasites, as well as any changes that might have been introduced during the propagation procedure. The read depth per genome was at least ×38 coverage, which was subjected to *de novo* assembly and compared to the most recent genome assemblies for *Pb* and *Pf*. *Pb*Vac sequencing results confirmed a single insertion site of the *Pf*CS gene and its flanking sequences in the *Pb*P230p gene present in chromosome 3 of the *Pb* genome (Fig. 2) and showed that no additional *Pf* genetic material is present in the transgenic parasite’s genome. *De novo* assembly of the different samples revealed no large rearrangements or deletions and no copy number variations were found between the transgenic and the reference sequences, except for the UTR of *Pb*UIS4. No differences were detected between the *Pb*Vac sequences prior to and after cyclical propagation and a single base change was detected between the wild-type and transgenic lines, which is consistent with the known overall *Plasmodium* mutation rate.^19^ Overall, these results establish the full genomic sequence of *Pb*Vac and confirm the insertion of the *Pf*CS gene in the parasite’s expected genomic locus. To confirm the stability of this insertion, PbVac MCB parasites underwent 8 passages through SPF Balb/c mice and 2 passages through mosquitoes, and the insert region of blood stages of the the PbVac parasite collected from selected mice was sequenced (Fig. S1). Our results demonstrate that the *Pf*CS gene remains correctly inserted in the expected locus throughout this procedure of mechanical passage (data not shown), indicating that no loss if genetic material is expected to occur during the 2 passages through mice and 1 passage through mosquitoes that must precede parasite administration in a clinical setting.

### High-sensitivity qRT-PCR detection and quantification of PbVac parasites

**Fig. 3:**
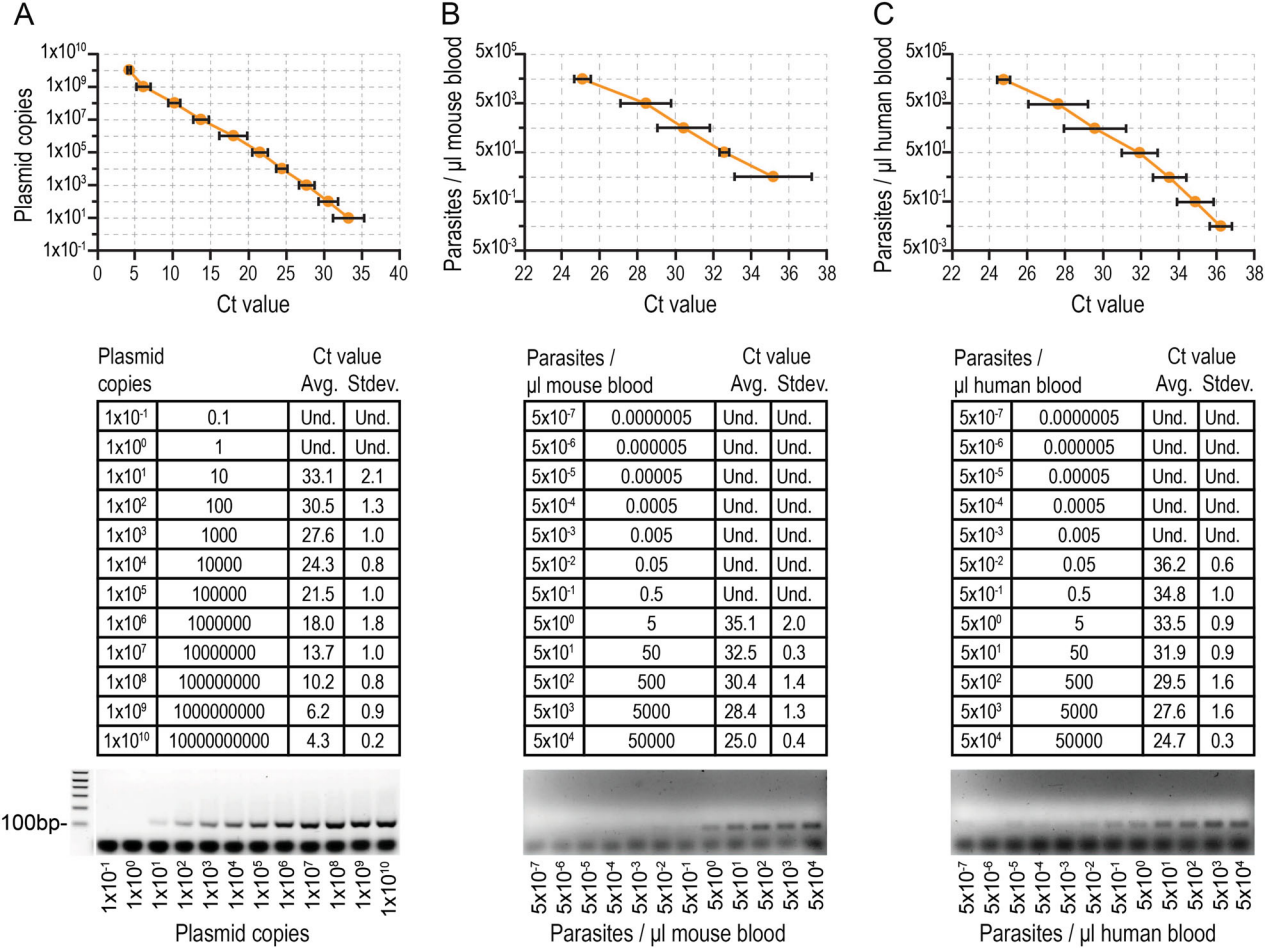
*Pb*Vac parasite detection and quantification by high-sensitivity qRT-PCR. Standard curves (top), Ct values (middle), and gel electrophoresis analysis (bottom) following qRT-PCR amplification of a 134 bp fragment of the *Pb*Vac 18S ribosomal gene in serial dilutions of a plasmid containing that fragment (**a**), of the *Pb*Vac parasite in mouse blood (**b**), and of the *Pb*Vac parasite in human blood (**c**). Dots indicate the average CT value and bars indicate the standard deviation obtained for at least three biological replicates

Clinical evaluation of the *Pb*Vac vaccine candidate requires close monitoring of the highly unlikely possibility of appearance of blood stage parasitemia in human subjects. To this end, we established a quantitative real-time PCR (qRT-PCR) assay that could adequately detect *Pb*Vac in different tissues, including human blood samples. The linear range of amplification of the TaqMan qRT-PCR and the assay’s limit of detection were determined employing tenfold serial dilutions of a plasmid encoding a fragment of the *Pb* 18S rRNA gene (Fig. 3a). The assay’s limit of detection was calculated at 0.0001 copies of target standard plasmid per PCR reaction, with linearity observed between 0.1 and 10^10^ copies of plasmid, and the efficiency of the assay was calculated at 113%. The dynamic range of the assay for detection of *Pb*Vac was initially determined in mouse blood using genomic DNA extracted from serial dilutions of synchronized *Pb*Vac-infected blood cultures (Fig. 3b). The calculated limit of detection of the assay was 0.006 parasites/μl of blood, with linearity observed between 6.1 and 61,674 parasites/μl of blood. Agarose gel electrophoresis analysis confirmed the presence of a single DNA band corresponding to the expected size of the amplicon of interest (134 bp) (Fig. 3b). The assay was applied to the detection of *Pb*Vac in human blood, yielding a limit of detection of 0.05 parasites/μl of blood. Analysis of the reaction products by agarose gel electrophoresis confirmed the presence of a single 134 bp DNA band (Fig. 3c). Our results show that this assay constitutes a highly sensitive, precise, and reproducible assay to detect and quantify *Pb*Vac on a small volume of whole blood with minimal pre-analytical processing to support clinical care and malaria vaccine research. Our results also establish 18S rRNA as a reliable target for a highly sensitive qRT-PCR assay, which allows the detection of as few as 0.05 parasites/μl of human blood.

### Distribution of PbVac in rabbit tissues

**Fig. 4:**
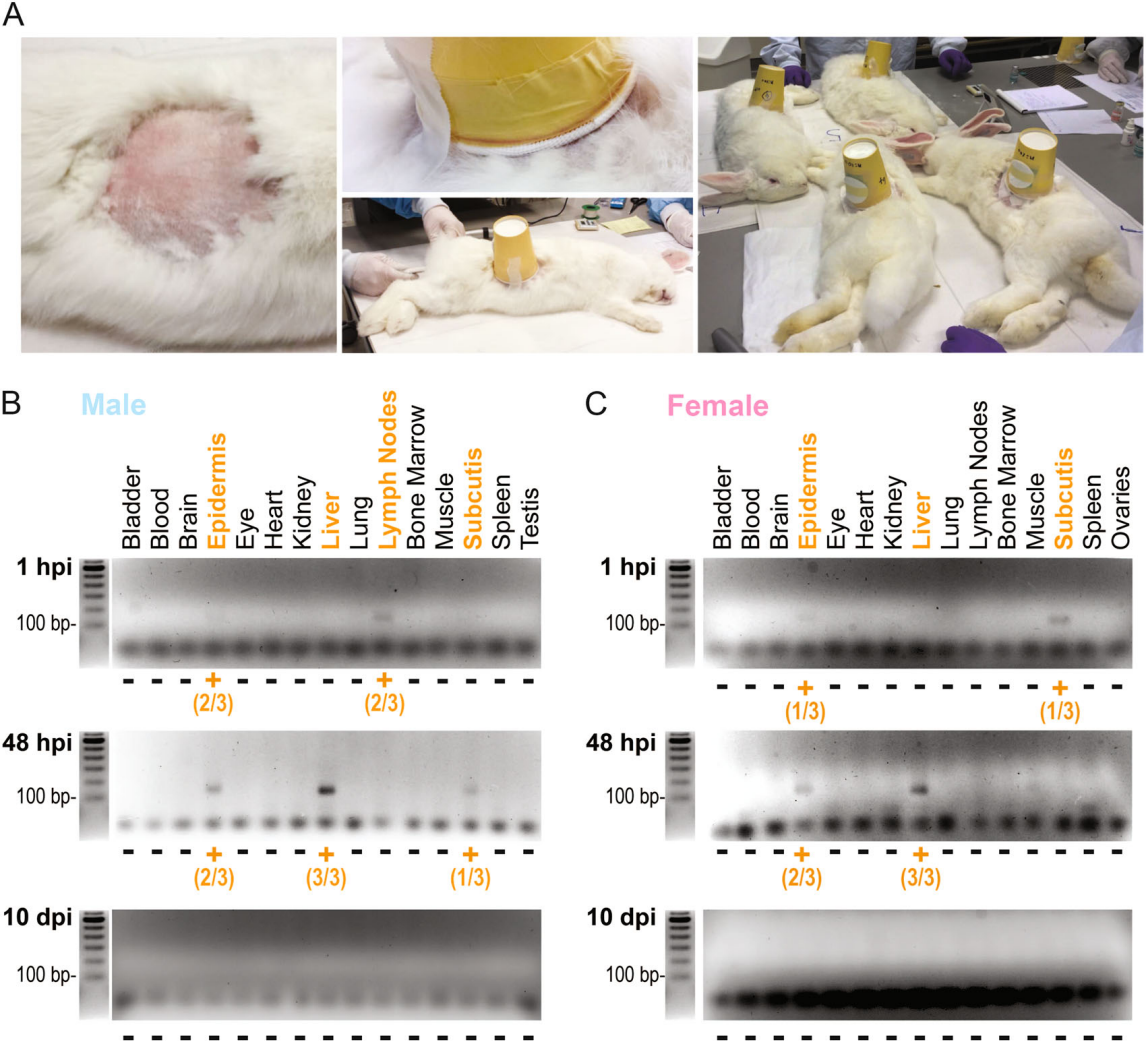
*Pb*Vac tissue biodistribution in NZW rabbits. **a** *Pb*Vac administration to rabbits by mosquito bite. Left: a circular area of ~10 cm diameter of the rabbit flank was shaved prior to exposure to mosquitoes. Middle: a container with 100 mosquitoes was placed on the shaved flank of the sedated animal and taped to the skin. Right: the mosquitoes were allowed to feed on the sedated animals for 15 min. **b**, **c** Gel electrophoresis analysis of products of qRT-PCR amplification of a 134 bp fragment of the *Pb*Vac 18S ribosomal gene in various organs of male (**b**) and female (**c**) rabbits at different time points after parasite administration. Orange highlights correspond to organs where *Pb*Vac was detected. The numbers correspond to the number of animals where the parasite was detected in a particular organ/the number of animals for which that organ was analyzed. Three biological replicates, each including one male and one female NZW rabbit, were employed per time point

Given the promiscuous nature of *Pb* parasites, safety requirements demand an investigation of the biodistribution of *Pb*Vac in a suitable animal model. Our previous report established that *Pb*Vac infects and develops in NZW rabbit hepatocytes but not in NZW rabbit red blood cells, mimicking the expected behavior of this parasite in humans.^18^ Thus, NZW rabbits were selected as the animal model of choice to assess the tissue distribution of *Pb*Vac in vivo. To this end, NZW rabbits were infected by the bites of 75 *Pb*Vac-infected *A. stephensi* mosquitoes (Fig. 4a) and killed 1 h, 48 h, and 10 days after infection. Bladder, blood, brain, epidermis, eye, heart, kidney, liver, lung, lymph node, bone marrow, muscle, spleen¸ subcutis, ovary, and testes samples were collected at each time point of euthanasia and processed for RNA extraction, followed by cDNA synthesis and qRT-PCR analysis using the methodology described above. RNA quality was confirmed by gel electrophoresis analysis of brain, heart, and lung samples (Fig. S2). The detection sensitivity of the 18S-*Pb* gene in the standard plasmid was shown to be unaffected by the presence of cDNA from brain, heart, and lung tissues (Fig. S3A). Additionally, effective extraction of parasite RNA in the presence of rabbit tissues was confirmed by qRT-PCR and agarose gel electrophoresis analyses (Fig. S3B). Our qRT-PCR analysis of organ samples collected 1 h after sporozoite administration revealed the presence of parasite DNA in the subcutis, epidermis, and lymph nodes at, or proximal to, the site of administration, whereas at 48 h post-infection (hpi), the parasite was detected in the epidermis and subcutis, as well as in the liver. Following the qRT-PCR analysis, all samples were analyzed by agarose gel electrophoresis. A band of the expected fragment size (134 bp) was observed for the samples yielding a positive signal on the qRT-PCR analysis. No parasites were detected in any of the organs collected 10 days post-infection (dpi) (Fig. 4b, c). Overall, our results show that the tissue biodistribution of *Pb*Vac follows a pattern compatible with the safety of its administration to humans and with the elicitation of a pre-erythrocytic immune response, as parasites are detected near the site of administration at 1 and 48 h after injection, as well as in the liver at the latter time point. Importantly, parasites are absent from any tissues 10 days after administration, indicating that the parasites are fully eliminated between 2 and 10 days after injection.

### Toxicological analyses of PbVac administration to rabbits

**Fig. 5:**
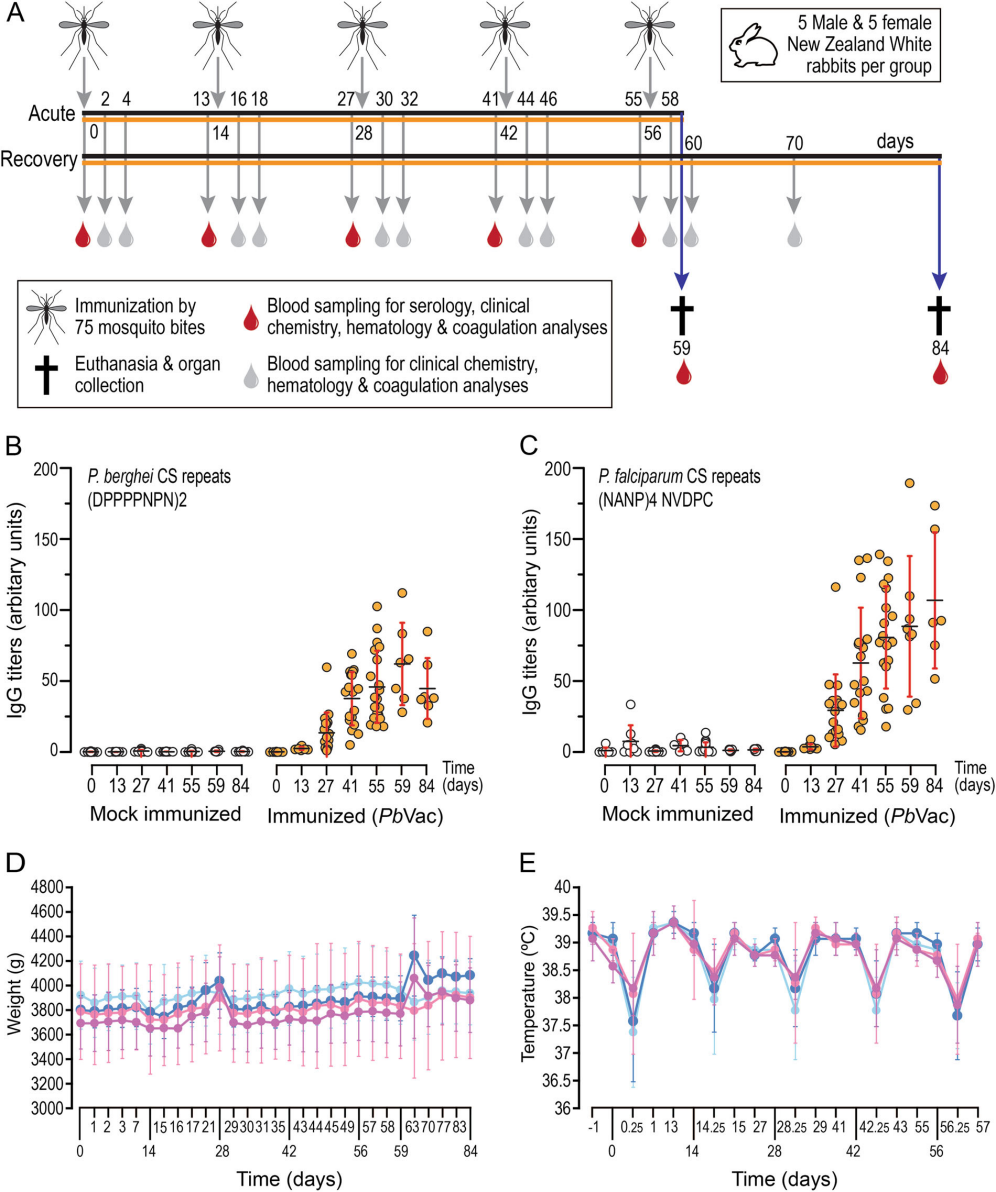
Toxicology of and humoral responses elicited by *Pb*Vac administration to NZW rabbits. **a** Schedule of parasite administration, sample collection, and killing (arrows) in rabbits subjected to the bites of 100 non-infected (black horizontal line, 5 male and 5 female NZW rabbits for each experimental group) or 100 *Pb*Vac-infected mosquitoes (orange horizontal line 5 male and 5 female NZW rabbits for each experimental group). **b**, **c** “Vaccine take” illustrated by the amount of anti-*Pb*CS repeats (**b**), anti-*Pf*CS repeats (**c**) antibodies detected in serum samples of mock-immunized and *Pb*Vac-immunized male and female rabbits, collected throughout the duration of the study (bars represent average of all observations per time point and respective standard deviation). **d** Body weight of male and female rabbits throughout the duration of the study. **e** Body temperature of male and female rabbits throughout the duration of the study. **d**, **e** Light and dark blue represent male rabbits subjected to non-infected mosquito bites or *Pb*Vac-infected mosquito bites, respectively, and light and dark pink represent female rabbits subjected to non-infected or *Pb*Vac-infected mosquito bites, respectively. Dots represent the average value of all animals within the group and bars indicate the standard deviation

In order to evaluate possible toxic effects of *Pb*Vac administration to a non-permissive host, a large toxicology study was conducted in NZW rabbits. The main study employed 40 animals subjected to 5 exposures to 97 non-infected or *Pb*Vac-infected mosquitoes, killed three (acute phase) or 28 (recovery phase) days after the last application of the mosquitoes (Fig. 5a). “Vaccine take” was confirmed by ELISA assessments of antibodies against *Pb*CS and *Pf*CS at different points after parasite administration (Fig. 5b, c). At in-life cage side observations, all animals showed normal water and food consumption patterns, and regular excretions of urine and feces. No effects of *Pb*Vac administration on clinical observations were noted during or after vaccination. In general, the body weights of individual rabbits showed some minor variations, which were correlated to blood sampling or vaccination and were considered to reflect stress during these processes. Animals recovered fast from the loss in body weight and quickly returned to their weight before the handlings. Overall, no significant body weight loss or gain was observed, indicating the tolerability of the test article (Fig. 5d). Body temperature remained within limits (no fever, defined as a temperature greater than 40 °C, was recorded) shortly after vaccination and throughout the next day. No increase in body temperature was observed 6 and 24 h after immunization. Rather, as a consequence of the sedation all rabbits experienced a decrease in body temperature 6 h after administration, which returned to normal 24 h later (Fig. 5e). Ophthalmological examination of the rabbits in the acute and recovery groups 1 day prior to their necropsies (day 58 and day 83, respectively) revealed no abnormalities. The analyses of serum, plasma and EDTA blood samples revealed transient trends in clinical chemistry, coagulation, and hematology parameters over time. The changes observed in these parameters were within the range of normal values and standard deviations were similar across groups within the study and thus considered non-adverse. Creatinine phosphokinase and lactate dehydrogenase levels on day 0 were increased and were above the normal range in both treated and control groups, which is attributed to handling and stress.^20^

There were no premature decedents in the study and animals were killed on day 59 (acute groups) or 84 (recovery groups), as planned. At necropsy, macroscopic examinations of the external surface of the body, all orifices, injection sites, the cranial, thoracic, and abdominal cavities and their contents were performed. No abnormalities were observed in the animals in the acute or recovery phase groups regarding fur, skin, and body openings. On the injection site, only very minimal skin reactions were visible 3 days after application of the mosquitoes. No enlarged lymph nodes nor abnormalities in kidneys, lungs, heart, liver, brain, reproductive organs, and other organs were found. In one female rabbit from the infected recovery group, some very slight hematomas were observed in the skin at the injection site. In the same animal, a very large gall bladder with abnormal shape was found, as well as gas formation in the gut, a cyst with clear fluid in one of the ovaries, and a slightly smaller brain. The histopathology did not reveal further insights for these findings, which were therefore considered non-related. A female rabbit from the uninfected recovery group and a female rabbit from the infected recovery group each had small cysts on one ovary. Finding cysts in the ovaries of these rabbits is expected for this species and age. Similar incidence in the control and treated groups indicate this is not likely an effect of *Pb*Vac, so the finding was considered non-related to *Pb*Vac. No clinically relevant differences in organ weights and no gross lesions were observed macroscopically. There was no *Pb*Vac-related alteration in the prevalence, severity, or histologic character of incidental microscopic findings recorded at histopathological examinations. No *Pb*Vac-related morphologic alterations were observed on day 59 (acute groups) or day 84 (recovery groups).

### Drug sensitivity of liver and blood stage PbVac parasites

Balb/c mice were used to assess sensitivity of *Pb*Vac parasites to the commonly used antimalarials chloroquine, Coartem^®^, and Malarone^®^ in vivo. Our results showed that both Malarone^®^ and Coartem^®^ completely eliminated blood stage parasites when treatment was initiated either when parasites became detectable or at 1.5% parasitemia (Table 1). Chloroquine treatment also abolished blood infection in both cases but recrudescence was observed when treatment was initiated when parasitemia reached 1.5% (Table 1). Collectively, our results show that both Malarone^®^ and Coartem^®^ can be employed to effectively eliminate *Pb*Vac’s blood forms in the very unlikely event of human blood stage infection by this parasite.

**Table 1:** *Pb*Vac parasite clearance following administration of clinically relevant schedules and doses of selected drugs to mice

Drug	Schedule (hpi)	Dose in humans (w)	Dose in mice (w/W)	Parasitemia at treatment initiation	Day of parasite clearance
	*t* = 0/6/24/48	*t* = 0–600 mg	*t* = 0–64 mg/kg^a^	Onset	3
Chloroquine		*t* = 6/24/48–300 mg	*t* = 6/24/48–32 mg/kg^a^	~1.5%	2
Coartem®	*t* = 0/8/24/36/48/60	80 mg ART	8.4 mg/kg ART^a^	Onset	3
		480 mg LMF	50 mg/kg LMF^a^	~1.5%	3
Malarone®	*t* = 0/24/48	1 g ATV	25 mg/kg ATV^b^	Onset	3
		400 mg PRG	10 mg/kg PRG^b^	~1.5%	3

*hpi* hours post-infection, *ART* artemether, *LMF* lumefantrine, *ATV* atovaquone, *PRG* proguanil

^a^Allometry-scaled dose

^b^¼ of allometry-scaled dose

Because Malarone^®^ is the prefered drug to use in the context of controlled human malaria infection (CHMI) studies and is able to clear liver stage parasites, we selected this drug to investigate its effect on *Pb*Vac liver infection. Our results showed that Malarone^®^ treatment completely abolished hepatic infection, measured 46 h after sporozoite injection, unlike the untreated control mice, which displayed significant liver parasite loads (Fig. S4). These results show that Malarone^®^ effectively eliminates *Pb*Vac from hepatic cells, in the unlikely event that they might persist in the liver following administration to human subjects. This effect is potentially due to Atovaquone, one of the active compounds of Malarone^®^, which is well known to kill hepatic stage *Pb* parasites.^21,22^

## Discussion

The clinical evaluation of vaccine candidates intended for human use, including WSp malaria vaccines, must meet strict regulatory requirements that ensure, to the fullest extent possible, the safety of the human subjects enrolled in their assessment. This study shows a comprehensive pre-clinical study of *Pb*Vac, obtaining the tools necessary for its use in clinical studies and revealing the evidence of its safety in non-permissive human hosts.

The first report of the use of *Pf* RAS in humans dates from 1973 and involved the administration of immunizing parasites by the bites of several hundred mosquitoes^7^. Since then, numerous clinical trials employing *Pf* RAS administered either by mosquito bite,^23^ subcutaneous or intradermal administration,^24^ or intravenous inoculation^8,25,26^ have been carried out. Likewise, the CPS immunization protocol has been evaluated in the clinic with parasites administered by mosquito bites,^15^ by intradermal injection,^27^ or direct venous inoculation.^28^ All of these clinical studies adhered to the regulations in place at the time when they were conducted, and all employed *Pf*, a malaria parasite whose natural hosts are human beings, as the immunizing agent. In this context, our recently proposed strategy for WSp vaccination, which relies on a genetically modified *Pb* parasite for immunization of humans against malaria,^18^ poses a whole new set of challenges that we have now addressed.

WSp vaccine candidates tested in the clinic have relied on human-infective *Pf* parasites, administered in the context of CHMI experiments.^29^ As *Pf* infections occur commonly in the field, the delivery of *Pf* sporozoites to humans in a clinical context can be considered a “natural” infection by a human-infective organism. *Pf*-based WSp vaccine candidates employed in these experiments are generated in mosquitoes infected by membrane feeding on parasite cultures maintained in in vitro conditions. However, the clinical evaluation of a rodent *Plasmodium*-based vaccine candidate constitutes uncharted territory and therefore demands a stringent pre-clinical safety assessment process. *Pb*Vac is not only a genetically modified organism but also one that requires passage through rodents before infection of mosquitoes to produce sporozoites. In order to enter clinical development, such parasites must be of known genetic identity and with no extraneous DNA and drug-resistance markers. *Pb*Vac was constructed using a double crossover homologous recombination method that ensures the permanent and stable introduction of the *Pf*CS gene into the *Pb* genome.^30^ The GIMO method of genetic modification employed further ensures the generation of a mutant parasite expressing a heterologous protein free of drug-resistance genes.^31^ In order to be used in humans, *Pb*Vac must not only be genetically stable but also microbiological contaminant free. Blood stage *Pb* parasites develop normally into merozoites in vitro but cannot egress from the erythrocyte without additional mechanical shear stress,^32^ hampering the infection of mosquitoes by standard membrane feeding assay technologies used for Pf, and demanding that the production of *Pb*Vac sporozoites for vaccination must rely on the infection of mosquitoes through biting *Pb*Vac-infected mice. We have thus created a MCB of *Pb*Vac, following the parasite’s cyclic propagation in SPF Balb/c mice and mosquitoes to remove any contaminants that might have arisen during prior manipulation of *Pb* and during the genetic modification procedures to produce *Pb*Vac. This MCB was extensively analyzed and shown to be free of microbiological contaminants. The determination of the parasite’s entire genomic sequence and the comparison with the *Pb* reference sequence^3^ further confirmed the correct modification of the parental parasite’s genome, as well as *Pb*Vac’s genetic homogeneity and stability.

Although *Pb* has been employed as a model for malaria research for decades, several gaps remain in our knowledge of its behavior in permissive and non-permissive mammalian hosts. *Pb* sporozoites are highly promiscuous and have been shown to infect a wide range of host cell types in vitro using both CD81-dependent and -independent invasion pathways.^33^ Thus, we have established a parasite detection method that enables assessing the presence of *Pb*Vac in a range of rabbit tissues, which ascertains *Pb*Vac’s tropism for liver cells in a non-permissive host in vivo. The clinical evaluation of the vaccine candidate is proposed to include 4 immunizations with *Pb*Vac, each delivered by 75 infected mosquitoes. Because of this, we performed an extensive evaluation of potential toxicity resulting from 5 consecutive administrations of *Pb*Vac delivered to rabbits by 97 infective mosquito bites each, ensuring 75 effective bites per administration. This study revealed the absence of toxicity as a result of vaccine administration, indicating the safety of its use in non-permissive human hosts.

The data presented here show that the parasite is completely eliminated from rabbits’ livers and all other organs analyzed up to 10 days after its administration. Additionally, our array of pre-clinical results has shown that *Pb*Vac is unable to lead to a patent blood stage infection in rabbits and is incapable of developing in human erythrocytes.^18^ Nevertheless, the use of *Pb*Vac in the clinic requires that safety measures are in place, in the unlikely event that the parasite should persist in the human subjects’ liver or lead to a patent blood stage infection in its human host. Malarone® has been used in malaria prophylaxis for nearly 2 decades.^34^ It is a combination of atovaquone and proguanil, two drugs that have been shown to display causal prophylactic activity directed against the liver stages of *Pf*^35^. Coartem^®^, which combines artemether and lumefantrine, has been shown to display high efficacy in the treatment of uncomplicated,^36^ acute,^37^ and multidrug-resistant^38^ *Pf* malaria, and to exert antimalarial activity in a mouse model of *Pb.*^39^ Our results show that administration of clinically relevant doses of Malarone^®^ effectively clears hepatic *Pb*Vac parasites, whereas clinically relevant doses of both Malarone^®^ and Coartem^®^ fully abolish *Pb*Vac blood stage parasitemia from infected mice. These results compound a solid “safety net” that ensures the complete elimination of *Pb*Vac from the patient organism in a clinical setting.

The present study constitutes a thorough assessment of a rodent malaria parasite intended for human use. Our findings elucidate novel aspects of the biology of *Pb* in permissive and non-permissive hosts, providing further support for the laboratory use of this parasite model. Crucially, they also address safety and regulatory concerns regarding the use of *Pb*Vac in the clinic, paving the way to the assessment of its safety and protective efficacy in Phase I/IIa clinical trials.

## Materials and methods

### Animals

All rodent experiments were carried out in male C57BL/6 or Balb/c mice (from Charles River Laboratories), aged 6 to 10 weeks. Animals used for blood passage were C57BL/6, older than 6 weeks. In each experiment, all animals were matched by age. New Zealand White (NZW) rabbits were also purchased from Charles River Laboratories. All animals were housed at the animal facilities of the Instituto de Medicina Molecular (iMM Lisboa), according to the guidelines of the Animal Care Committee of iMM Lisboa (ACCiMM). All protocols involving live animals were approved by the ACCiMM.

### Parasites

*Pb* ANKA cl15cy1 was selected as the wild-type *Pb* (WT-*Pb*) line in the current study. This parasite line derives from an isolate originally obtained from *Anopheles dureni millecampsi* mosquitoes caught in the Democratic Republic of Congo in 1965.^40^ *Pb* ANKA cl15cy1 was employed to generate the *Pb* ANKA line 1596cl1,^31^ which served as the motherline for the generation of *Pb*Vac^18^ by the ‘gene insertion / marker out’ (GIMO) method. ^31^

### Generation of master cell bank (MCB)

To generate a MCB of *Pb*Vac-infected red blood cells (iRBC), specific pathogen free (SPF) Balb/c mice were infected by intraperitoneal injection of 1.5 × 10^6^ cryopreserved wild-type *Pb*- or *Pb*Vac-iRBC. Parasitemia and parasite exflagellation were monitored daily. These mice were then employed to infect *Anopheles stephensi* mosquitoes through 2 consecutive days of mosquito bites. At ~7% parasitemia, blood was collected by heart puncture, aliquoted and cryopreserved in liquid nitrogen until further use. These vials contained *Pb*Vac-infected iRBC prior to cyclical propagation, used in subsequent whole-genome sequencing studies (see below). Twenty-one days after the blood meal, *Pb*Vac-infected *A. stephensi* mosquitoes were allowed to feed on three naive SPF Balb/c mice. Parasitemia and parasite exflagellation were monitored daily and these were then employed to infect mosquitoes through two consecutive days of mosquito bites. At ~6.5–8.5% parasitemia, blood was collected by heart puncture, aliquoted and cryopreserved at −80 °C. This procedure was repeated another two times, for a total of three passages of the *Pb*Vac parasites through mosquitoes and four passages through SPF Balb/c mice. Following the last passage through SPF Balb/c mice, blood was collected by heart puncture when parasitemia was ~6.5%, aliquoted and cryopreserved at −80 °C. Collected blood was further employed to infect 50 Balb/c mice by intraperitoneal injection of *Pb*Vac-iRBC. When parasitemia in these mice reached ~3%, the mice were killed and the blood was collected by heart puncture and pooled. Two aliquots of this blood were collected for microbiological analyses and whole-genome sequencing (see below). The remaining blood was aliquoted and cryopreserved in liquid nitrogen, constituting *Pb*Vac iRBC MCB (Fig. 1). The parental wild-type *Pb* parasites underwent a similar procedure as that described for *Pb*Vac, enabling a comparison of the genome sequences of both parasites before and after cyclical propagation (see below). All Balb/c mice employed in this study were purchased from Charles River Laboratories and had a certified SPF health status.

### Microbiological analyses of MCB samples

The final batch of mice employed in MCB generation were further tested for the full Federation for Laboratory Animal Science Associations (FELASA) panel for SPF mice.^41^ One random mouse was sampled for every other cage of 5 mice (5 samples from 10 cages). From each sampled mouse, 2–3 drops of blood were collected onto a paper sampling kit. Samples were analyzed by IDEXX BioResearch using the FELASA 2014 Serology Panel, which includes serologic evaluation for antibodies to *Clostridium piliforme*, *Mycoplasma pulmonis*, Ectromelia, EDIM, LCMV, MAV1, MAV2, MHV, MNV, MPV, MVM, PVM, REO3, Sendai, and TMEV (Table S1). Mouse fecal pellets, oral swabs, and fur swabs were collected and tested in pools of samples from 10 animals, as allowed per test sensitivity, according to the FELASA 2014 Bacteriology + Parasitology Panel (Beta Strep Grp A PCR, Beta Strep Grp B PCR, Beta Strep Grp C PCR, Beta Strep Grp G PCR, *C. rodentium* PCR, *C. kutscheri* PCR, *C. piliforme* PCR, *Helicobacter* genus, *M. pulmonis* PCR, *P. pneumotropica*-Heyl PCR, *P. pneumotropica*-Jawetz PCR, *Salmonella* Genus PCR, *S. moniliformis* PCR, *S. pneumoniae* PCR, *Cryptosporidium* PCR, Entamoeba PCR, Giardia PCR, Mite PCR, Pinworm PCR, and *Spironucleus muris* PCR) by Charles River Laboratories (Table S1). Finally, MCB samples were tested for possible human pathogens and adventitious viruses, including arboviruses that could be transferred from the mosquitoes. These analyses were performed by Charles River Laboratories and included the Human Comprehensive CLEAR Panel [Adeno-associated virus, LCMV PCR, Human cytomegalovirus, HANT (Hantavirus Hantaan) PCR, SEO (Hantavirus) PCR, Hepatitis B virus, Hepatitis C virus, Epstein-Barr Virus, Herpesvirus type 6, Herpesvirus type 7, Herpesvirus type 8, Mycoplasma Genus PCR, HPV-16, HPV-18, Parvovirus B19, Hepatitis A virus, John Cunningham virus, BK virus, Human Foamy Virus, Human T-lymphotropic virus, HIV-1, and HIV-2] (Table S2) and detection assays of adventitious viruses by analysis of cytopathic effects (CPE), hemadsorption, and hemagglutination in 28 day assays. These assays employed Vero (African green monkey kidney, ECACC 84113001), MRC-5 (human embryonic lung, ATCC CCL-771), NIH 3T3 (Swiss mouse embryo tissue, ECACC 93061524), and BHK-21 (baby hamster kidney, ATCC CCL-10) indicator cell lines (Table S2), and PI3 (parainfluenza virus type 3, ATCC VR-93), H1N1 virus (influenza A virus, ATCC VR-219), Sindbis virus (ATCC VR-68), and vesicular stomatitis virus (VSV, ATCC VR-158) as control viruses, respectively. The suitability of the test method for its intended use was demonstrated in generic validations according to International Council for Harmonisation of Technical Requirements for Pharmaceuticals for Human Use (ICH) guidelines.

### Whole-genome sequencing analyses

The whole genomes of wild-type *Pb* and *Pb*Vac collected prior to or following cyclical propagation construction were sequenced at The Sanger Institute, UK. Parasite DNA was extracted by phenol/chloroform extraction following removal of leukocytes using a CF 11 column. The samples were prepared as noPCR library.^42^ They were sequenced on a MiSeq with 150 bp reads, aiming for a fragment length of 500 bp. The obtained reads were aligned with Burrows-Wheeler Aligner’s Smith-Waterman Alignment (BWA-SW)^43^ (parameter ‐a 1000, version 0.7.12 ‐r1039) and Bowtie2^44^ (-X1000‐very‐sensitive ‐N 1 ‐L 31 ‐rdg 5,2, version 2.1.0). The latest version of *Pf* 3D7 (version 3) and *Pb* ANKA (version 3) genomes from GeneDB,^3,45^ July 2015 version, were used as references. PCR duplicates were flagged with Picard. For variant calling, the bam files for the different samples were merged, realigned with GATK realign, and variants were called with GATK variant caller “UnifiedGenotyp”. *De novo* assembly was performed with Velvet^46^ (parameter k‐mer: 81 (sample PbMP8 k-mer of 61 ‐exp_cov auto‐ins_length 350 ‐ins_length_sd50 ‐cov_cutoff 5 ‐min_contig_lgth 500 ‐min_pair_count 10; Version 1.2.07). Contigs were ordered and orientated against the *Pb* genome using ABACAS2.^47^ Potential insertion sites (including the target gene), potential recombination “not-proper paired” reads, as well as differences between *de novo* assemblies, were searched for. Detection of potential copy number variation (CNV) was performed manually through the evaluation of changes in the read coverage.

### Mouse infection and parasite synchronization for parasite detection assays

All mice used in the parasite detection assays were infected intraperitoneally (i.p.) with *Pb*Vac, either with 1 × 10^6^ fresh, infected red blood cells (pRBCs) collected from a passage mouse previously infected from a frozen vial (blood passage), or with 2 × 10^6^ frozen pRBCs (infection with frozen vial). Parasitemia in the inoculum (number of pRBCs / total number of RBCs x 100) was assessed by microscopic evaluation of Giemsa-stained tail-blood smears, by counting at least 2000 RBCs.

*P. berghei* is one of the few mammalian parasites whose schizonts do not rupture spontaneously in vitro. This feature is the rational support behind the parasite synchronization protocol described below, based on,^48^ which results in most of the pRBCs being synchronized in the more mature stages after overnight (o/n) incubation. Briefly, infected mice were killed by CO_2_ inhalation and blood was collected by cardiac puncture, into 30 ml of PBS. Blood was then centrifuged at 650×*g* (without brake), for 10 min at 4 °C. The supernatant was discarded and the pellet carefully resuspended in 50 ml of the culture medium solution (RPMI 1640, supplemented with 25% FBS, 2mM l-Glutamine, penicillin–streptomycin at 50 μg/ml, 1% (v/v) HEPES, 1% (v/v) non-essential amino-acids), before being divided into two 25 cm^3^ culture flasks. Flasks were incubated at 37 °C under an atmosphere of 5% O_2_, 5% CO_2_, 90% N_2_ for 12–16 h. After o/n incubation, mature pRBCs were isolated by a density gradient. Briefly, the RBC suspensions were carefully pipetted on top of 10 ml of a 65% (v/v) Nicodenz solution and then centrifuged at 550×*g*, for 30 min at 21 °C (without brake). Most of the upper layer was discarded and the interface between the upper layer and the bottom layer was pipetted into a 50 ml Falcon tube and PBS was added up to a volume of 40 ml. This cell suspension was centrifuged at 580×*g*, for 8 min (without brake) and the pellet was resuspended in 400 μl of PBS 1×. Purified schizonts were then injected intravenously into the tail veins of 2–3 mice. Infected blood was collected either 3–4 h after injection of the schizonts (ring forms from the first cycle) or 24–26 h after injection of the schizonts (ring forms from the second cycle). Parasitemia and percent of synchronization (number of rings/ total number of parasites × 100) was then assessed.

### DNA extraction for parasite detection assays

Collected blood was washed three times with 20 ml of RNAse/DNAse-free PBS. The supernatant was discarded and the pellet was resuspended in 500 μl PBS. Parasiteamia was counted as described before; the number of total RBCs was counted in a Neubauer chamber.

A series of tenfold dilutions of the infected blood in uninfected blood from healthy mice with known baseline erythrocyte counts (around 4–5 × 10^6^ RBC/μl cell suspension) was prepared. The assay was then applied to the detection of *Pb*Vac in human blood. To this end, *Pb*Vac-infected RBCs were serially diluted in naive human blood and genomic DNA was extracted and analyzed. Genomic DNA was isolated from 20 μl of blood from every sample in the dilution series using the DNAeasy Blood and Tissue Kit (Qiagen, Hilden, Germany) according to the manufacturer’s instructions, following the Nonnucleated blood protocol. The DNA samples were dissolved in 50 μl of elution buffer.

### Parasite detection by quantitative real-time PCR

Parasite detection employed *P. berghei* 18S rRNA-specific primers. A fragment of the *P. berghei* gene encoding the 18S ribosomal RNA subunit was amplified by quantitative real-time PCR (qRT-PCR) using TaqMan chemistry and hydrolysis probes. Probes were labeled with 6-carboxy-fluorescein (FAM) and with minor groove binding non-fluorescent quencher (MGB). For the qRT-PCR reaction, 4 μl of sample DNA was used along with 250 nM probe, 300 nM of each primer, water, and 10 μl TaqMan Universal Master Mix (Applied Biosystems, USA), in a total reaction volume of 20 μl. The sequences for primers and TaqMan probe used are listed in Table S3. Reactions were performed on the ABI Prism 7500 system (Applied Biosystems) with the following cycling conditions: 50 °C for 2 min, initial denaturation at 95 °C for 10 min, then 40 cycles at 95 °C for 15 s, and 60 °C for 1 min. Fluorescence data was collected during the annealing/extension step at 60 °C. All qRT-PCR reactions were prepared in a laminar flow hood with filtered pipette tips. All tests were performed in duplicate, and samples were tested on plates that included appropriate negative (reaction mixture without DNA and with DNA extracted from a healthy mouse) and positive (plasmid and a known positive sample) controls. External standardization was performed using plasmids encoding the full-length 18S rRNA gene cDNA cloned in TOPO TA (Invitrogen).^49^ The concentration of plasmid DNA was determined by spectrophotometry for the calculation of the DNA copy number. For standard curve analysis in real-time PCR assays, the plasmid was serially diluted tenfold in nuclease-free water from 10^10^ copies to 10^-5^ copies per qRT-PCR reaction. The efficiency (*E*) of the assay was calculated using the equation *E* = (10^(−1/slope)^) − 1 × 100.

### Infection and sample collection for tissue distribution assays

NZW rabbits (5 rabbits per group; 3 males and 2 females) were infected by the bites of 75 *Pb*Vac-infected *A. stephensi* mosquitoes and killed at 1 h, 48 h, and 10 days after infection. One control group (3 rabbits) of non-infected rabbits was killed at the same time as the rabbits killed 10 days after infection. The following tissues were collected and flash frozen in liquid nitrogen until further processing: bladder, blood, brain, epidermis (at the site of administration), eye, heart, kidney, liver, lung, lymph node (proximal to the site of administration), bone marrow, muscle (at the site of administration), spleen, subcutis (at the site of administration), ovaries, and testes.

### Analysis of tissue distribution samples

A sample of each tissue was homogenized in denaturing solution (4 M guanidine thiocyanate, 25 mM sodium citrate [pH 7], 0.5% N-lauroylsarcosine in diethyl pyrocarbonate [DEPC]-treated water) supplemented with 0.1 M β-mercaptoethanol) using a minibead beater for tissue disruption. RNA was extracted using TRIzol® (Ambion by Life Technologies), or TRIzol®LS (Ambion by Life Technologies) for blood samples, according to the manufacturer’s instructions. After extraction, DNase I treatment was performed with NzyTech’s DNase, according to the manufacturer’s instructions. RNA was quantified using a NanoDrop 1000 spectrophotometer and cDNA was synthesized using the NZYTech First-Strand cDNA synthesis kit. A fragment of the *P. berghei* gene encoding the 18S ribosomal RNA subunit was amplified by qRT-PCR using TaqMan chemistry and hydrolysis probes. The qRT-PCR reaction was performed in a total volume of 10 μl, containing 2 μl of sample cDNA, 250 nM TaqMan Probe (1 μl), 300 nM of each primer (1 μl), RNase/DNase-free water (1 μl) and 5 μl TaqMan Universal Master Mix (Applied Biosystems, USA). The sequences for primers and TaqMan Probe used are listed in Table S3. Reactions were performed on a ViiA 7 system (Applied Biosystems) with the following conditions: 50 °C for 2 min, initial denaturation at 95 °C for 10 min, then 40 cycles at 95 °C for 15 s, and 60 °C for 1 min. Fluorescence data was collected during the annealing/extension step at 60 °C. All experiments were performed in duplicate and samples were tested on plates that included appropriate negative (reaction mixture with RNase/DNase-free water instead of DNA) and positive (plasmid DNA and infected mouse livers at 48 h post-infection) controls. External standardization was performed as described above using tenfold serial dilutions of plasmid in nuclease-free water from 10^10^ copies to 10^-2^ copies per qRT-PCR reaction. The efficiency of the assay was calculated as described above. The sensitivity of detection of the standard plasmid in the presence of brain, heart, and lung tissue was further confirmed by spiking samples containing 2 out of 20 μl of cDNA previously synthesized from 1 μg RNA extracted from rabbit brain, heart, and lung tissues, and already identified as TaqMan-negative for this gene, with five dilutions of the plasmid (10^3^, 10^2^, 10^1^, 10^0^, 10^-1^) prior to qRT-PCR and agarose gel electrophoresis analyses. To confirm the effectiveness of the extraction of parasite RNA in the presence of rabbit tissues, 10^4^ *Pb*Vac sporozoites were added to 200 μl homogenates of rabbit brain, heart, and lung tissues, previously shown to be TaqMan-negative for 18S-*Pb*. Total RNA was extracted from the mixture and used to synthesize cDNA, which was analyzed by qRT-PCR using 18S-*Pb*-specific primers and by agarose gel electrophoresis. In each agarose gel image presented in the manuscript and Supplemental Information, all samples derive from the analysis of the same biological specimen and were processed in parallel.

### Toxicology assays

A toxicology study was performed by the Radboud University Medical Center and BioXpert B.V. in The Netherlands, under a project license granted by the Central Committee for Animal Experiments (CCD in Dutch) and the Animal Experiments Committee (DEC) consult (project license AVD905002015183). The study was conducted in 24-week-old male and female NZW rabbits (Envigo). Animals were SPF at delivery and were kept to maintain SPF status through monitoring and confirmation by microbiological health screening. All mosquitoes employed in the study were *A. stephensi* produced at the Radboud University Medical Center in Nijmegen, and were transported to the BioXpert B.V. Prior to immunization by mosquito bites, animals were sedated with medetomidine (Dexdomitor) and the application sites (i.e., flank) were shaved. A pilot study was initially performed in 5 animals subjected to the bites of 100 wild-type *Pb*-infected mosquitoes, to determine the mosquito feeding rate and to ascertain the impact of the medetomidine sedation on the latter. Our results showed that the mosquito feeding rate was 80.5% and was unaffected by medetomidine, whereas the mosquito mortality rate on the immunization day itself was estimated at ~4%. Thus, it was determined that the containers on the main study should contain 97 mosquitoes in order to attain the target dosage of 75 mosquito bites per immunization proposed for the evaluation of *Pb*Vac in the main study. Forty rabbits, 19 males and 21 females, were randomly assigned to the “infected” group or “uninfected, control” group for a total of 5 exposures to the bites of 97 *Pb*Vac-infected mosquitoes or 97 non-infected mosquitoes per rabbit, respectively, at Study Days (SD) 0, 14, 28, 42, and 56. Blood samples were collected from the marginal ear veins or saphenic veins without sedation. Samples for immunological analyses were collected on the day of each immunization and at euthanasia; samples for clinical chemistry, hematology, and coagulation analyses were collected on the day of immunization, 2 and 4 days after immunization, and at euthanasia. Animals were killed 3 (acute phase) or 28 (recovery phase) days after the last immunization with the mosquitoes. During the in-life phase, the effect of *Pb*Vac was evaluated by cage side observations, clinical examinations, body weight and daily food intake, body temperature, dermal draize observation, and ophthalmologic examination. The evaluations performed at necropsy included macroscopic examinations of the external surface of the body, all orifices, injection sites, the cranial, thoracic, and abdominal cavities, and their contents; hematology of bone marrow; determination of organ weights; and macroscopic and microscopic examination of putative gross lesions. Full panels of clinical chemistry and hematology on collected samples were performed by IDEXX Europe B.V. (Hoofddorp, The Netherlands). The post-mortem histopathological evaluation of organs was performed by Charles River Laboratories Den Bosch B.V. (‘s-Hertogenbosch, the Netherlands).

Statistical analyses were conducted to evaluate effect of immunization with infected mosquitoes. For body weight, body temperature, and dermal draize analyses, a Shapiro–Wilk normality test was initially performed to assess whether or not data were normally distributed. Body weight and body temperature were analyzed using a one-sample *t*-test, compared to the mean body weight on day 0 and mean body temperature on day −1 (i.e., 39.2 °C). Dermal draize scores were analyzed using a Wilcoxon signed-rank test. For analyses of clinical chemistry, coagulation, and hematology data, boxplots of all relevant parameters were made. Values of parameters that were below the detection limit were excluded (i.e., values of cholesterol, gamma-glutamyl transpeptidase, and total bilirubin). Subsequently, outliers per group were identified and excluded. Statistical comparison has been conducted by one-way analysis of variance (ANOVA) on multi-groups followed by Dunnett’s multiple comparison test if a significant difference was discerned. Furthermore, it was assessed whether or not the mean ± SD per time point was outside the normal range for rabbits. All statistical analyses have been conducted as two-sided tests with a level of significance of *p* < 0.05. For analyses of organ weights, the mean ± SD per organ was compared to their respective control using one-way ANOVA and/or *t*-test.

### ELISA

High protein-binding capacity 96 well enzyme-linked immunosorbent assay (ELISA) plates (Nunc MaxiSorp™ flat-bottom) were coated with synthetic peptide (Sigma) based on the repeat region of the *Pf*CS protein with the amino acid sequence (NANP)4NVDPC, or the repeat region of the *Pb*CS protein with the amino acid sequence (DPPPPNPN)2. The peptide was coated overnight at 4 °C at a concentration of 5 µg/ml in a volume of 50 µl per well. Plates were washed three times with *PB*S containing 0.1% (v/v) Tween-20 and blocked with 200 µl *PB*S containing 0.1% (v/v) Tween-20 and 1% (w/v) BSA for 30 min at room temperature. Plates were washed one additional time and samples serially diluted in PBS containing 0.1% (v/v) Tween-20 and 1% (w/v) BSA were added and incubated at 22 °C for 2 h. After washing four times, horseradish peroxidase-labeled goat anti-rabbit IgG (GE Healthcare UK) was added at a dilution of 1:2000 and incubated at 22 °C for 1 h. BD OptEIA™ TMB Substrate Reagent was then added for development and incubated for 1 to 3 min at 22 °C before stopping the reaction by adding 50 µl Stop solution (2 N H_2_SO_4_). The Optical density was determined using a microplate reader (Infinite M200). To serve as a positive control and to allow comparison between samples from different assays, a standard titration curve of at least 8 points, starting a dilution of 1/20 of a pool of rabbit sera from all immunized animals, was used as reference in all assays.

### Drug sensitivity assays

Drug sensitivity assays employed clinically relevant drug doses and schedules of administration as defined by the Centers for Disease Control and Prevention’s Guidelines for Treatment of Malaria in the United States for Malarone^®^ and chloroquine (http://www.cdc.gov/malaria/resources/pdf/treatmenttable.pdf), and by Novartis’s Highlights of Prescribing Information for Coartem^®^ (https://www.pharma.us.novartis.com/product/pi/pdf/coartem.pdf). Drug doses in mice were calculated based on allometric scaling, employing the formula: Allometric-scaled dose in humans = mice dose * (weight human/weight mice)^0.75^ ^50^

To avoid atovaquone toxicity in mice, ¼ of the allometric-scaled dose of Malarone^®^ was employed in the study.

For liver stage drug sensitivity assays for Malarone^®^, C57Bl/6 mice were infected by the bites of 20 *Pb*Vac-infected *A. stephensi* mosquitoes, followed by 3 administrations at 18-h intervals of the same dose of Malarone as that employed in the blood stage-clearance study (see below). *Pb*Vac-infected mice employed as controls were left untreated. Forty-six hours after sporozoite administration, mice were killed and liver parasite load was assessed by qRT-PCR as previously described.^51^

Blood stage drug sensitivity assays for Malarone^®^, chloroquine, and Coartem^®^ were performed on Balb/c mice. Human doses, allometry-scaled mouse doses, and administration schedules employed, are listed in Table 1. Mice were infected by intraperitoneal injection of 2 × 10^6^ *Pb*Vac-infected red blood cells. Blood parasitemias were monitored daily by microscopy analysis of Giemsa-stained blood smears. Treatment was initiated at two distinct moments, in different groups of mice, either at patency, as determined by blood smear analysis, or at ~1.5% parasitemia, as determined by blood smear analysis. Compounds were administered per os by oral gavage. Negative control mice were treated with an equivalent amount of drug vehicle, administered in a schedule equivalent to that of the drugs of interest. Positive control mice were treated by daily intraperitoneal injection of 0.7 mg chloroquine. During and after treatment, disease symptoms and parasitemia were monitored daily. If a treatment cleared the parasites from circulation, parasitemia was monitored for an additional 30 days until parasite clearance could be definitely established.

## Electronic supplementary material

Supplemental Material

## Data Availability

The data sets generated during and/or analyzed during the current study are available from the corresponding author on reasonable request. The accession numbers of the raw sequencing reads are ERS682077-ERS682080 (European Nucleotide Archive - https://www.ebi.ac.uk/ena).

## Electronic supplementary material

**Supplementary information** accompanies the paper on the *npj Vaccines* website (10.1038/s41541-018-0091-3).
