# Supplementary material for: Pre-clinical evaluation of a P. berghei-based whole-sporozoite malaria vaccine candidate
Source: NPJ Vaccines. 2018 Nov 27;3:54. doi: 10.1038/s41541-018-0091-3 (PMC6258718; doi:10.1038/s41541-018-0091-3)

Supporting Information for

Pre-Clinical Evaluation of a *P. berghei*-Based Whole-Sporozoite Malaria Vaccine Candidate

António M. Mendes, Isaie J. Reuling, Carolina M. Andrade, Thomas D. Otto Marta Machado, Filipa Teixeira, Joana Pissarra, Nataniel Gonçalves-Rosa, Dolores Bonaparte, João Sinfrónio, Mandy Sanders Chris J. Janse, Shahid M. Khan, Chris I. Newbold, Matthew Berriman Cynthia Lee, Yimin Wu, Christian F. Ockenhouse, Robert W. Sauerwein, Miguel Prudêncio

*correspondence to: mprudencio@medicina.ulisboa.pt

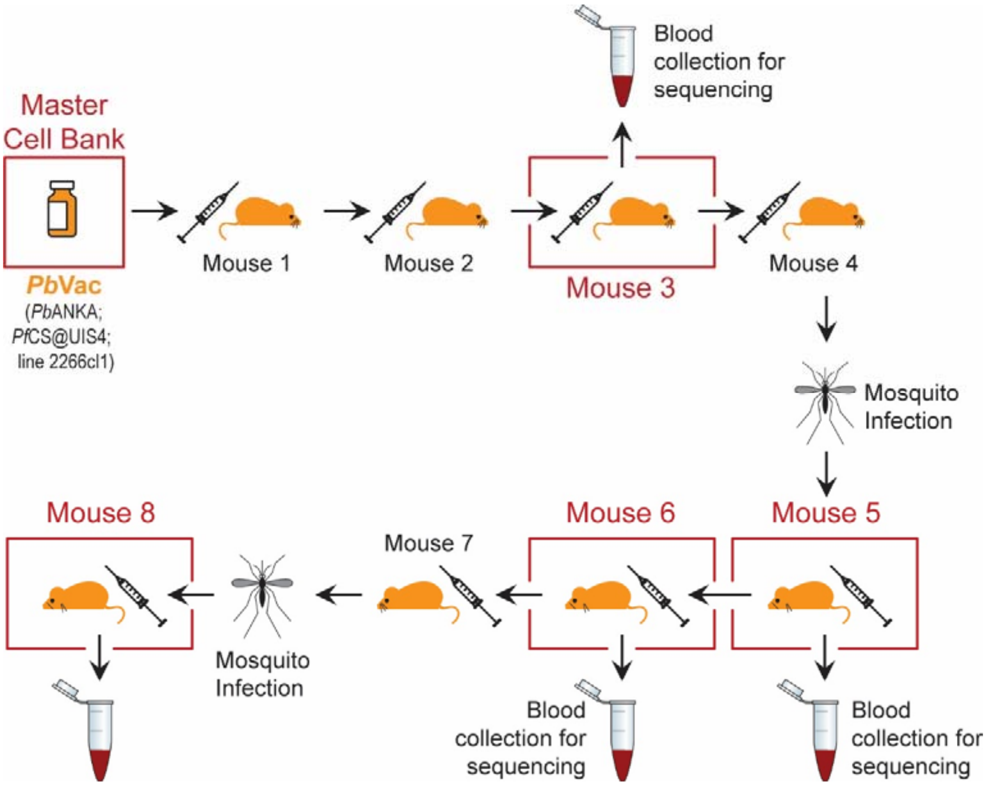

Fig S1. Assessment of insert stability in the *Pb*Vac parasite. Schematic representation of the sequential passage of MCB *Pb*Vac parasites through mice and mosquitoes. Red indicates the steps of blood collection for parasite sequencing.

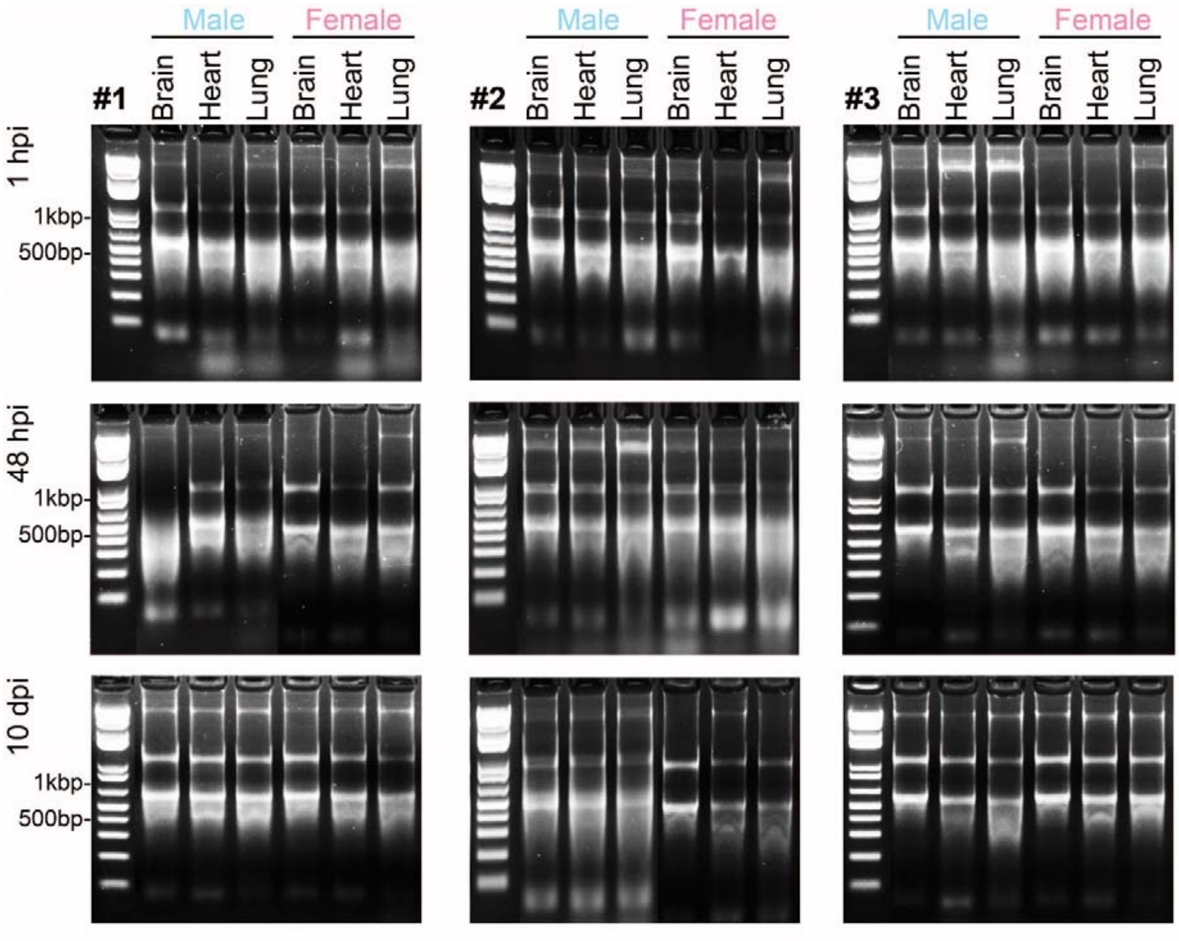

Fig S2. Representative gel electrophoresis analyses of the quality of RNA samples extracted from selected male and female rabbit organs at different times after parasite administration.

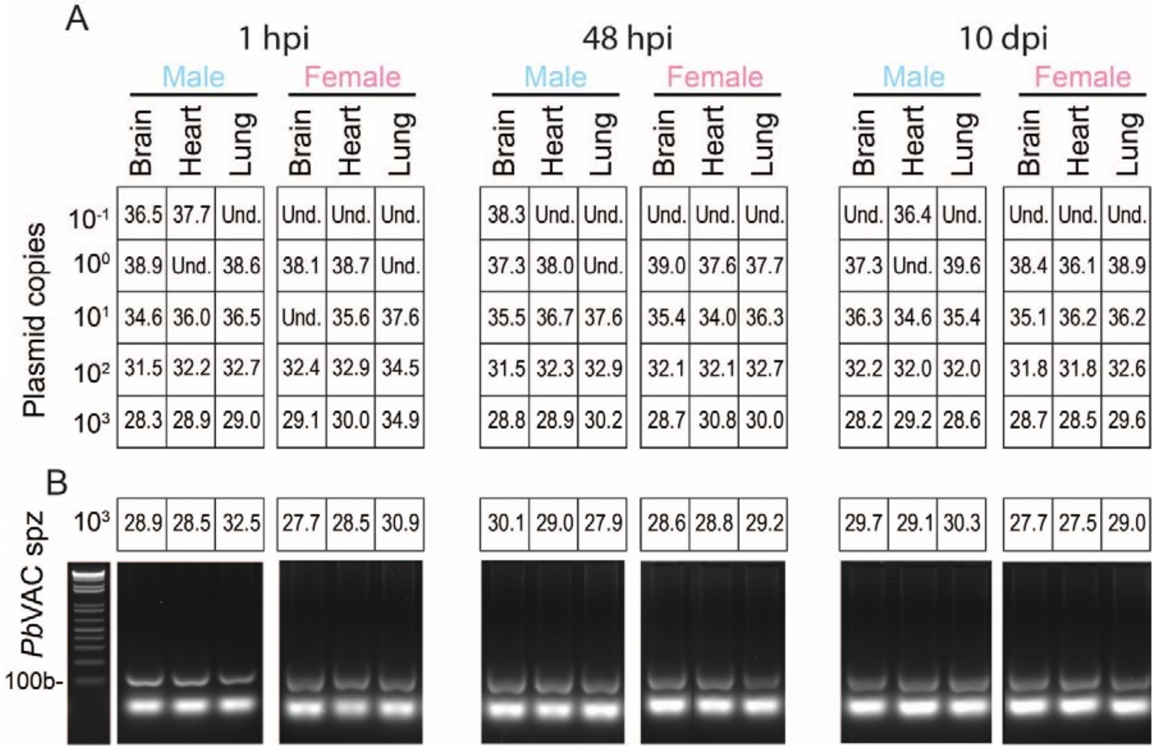

Fig S3. Detection of *Pb*Vac 18S ribosomal RNA extracted in the presence of selected "spiked" rabbit tissues. (**A**) qRT-PCR analysis of male and female rabbit tissue samples "spiked" with various amounts of a plasmid containing a fragment of the *Pb*Vac 18S ribosomal DNA gene. The numbers inside the squares represent the Ct values obtained following the qRT-PCR reaction. (**B**) qRT-PCR analysis of male and female rabbit tissue samples "spiked" with 10^3 *Pb*Vac sporozoites. Top: Ct values obtained following the qRT-PCR reaction; bottom: gel electrophoresis analysis of qRT-PCR amplification products. Collectively, these results demonstrate that *Pb*Vac can be efficiently extracted in the presence of rabbit tissue material and subsequently detected by qRT-PCR.

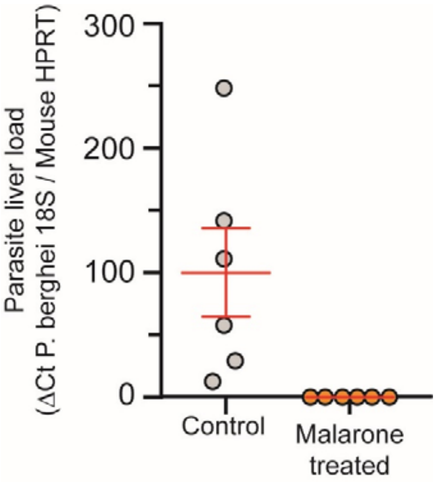

Fig. S4. Elimination of *Pb*Vac from mouse livers following Malarone® treatment. Liver *Pb*Vac burden of mice infected through the bites of 20 *Pb*Vac-infected mosquitoes and either left untreated (grey) or treated with 3 clinically relevant doses of Malarone® (orange), measured by qRT-PCR 46 hours after parasite administration.

Table S1 - ***Analyses on animal samples***

	Microorganism	Positive/Total
**FELASA 2014 Annual Serology Mouse Panel (IDEXX BioResearch)**	*Clostridium piliforme*	0/10
	Mycoplasma pulmonis	0/10
	Ectromelia	0/10
	EDIM	0/10
	LCMV	0/10
	MAV1	0/10
	MAV2	0/10
	MHV	0/10
	MNV	0/10
	MPV	0/10
	MVM	0/10
	PVM	0/10
	REO3	0/10
	Sendai	0/10

	Microorganism	Positive/Total
**FELASA 2014 Bacteriology + Parasitology Panel (Charles River)**	Beta Strep Grp A PCR	0/5
	Beta Strep Grp B PCR	0/5
	Beta Strep Grp C PCR	0/5
	Beta Strep Grp G PCR	0/5
	C. rodentium PCR	0/5
	C. kutscheri PCR	0/5
	C. piliforme PCR	0/5
	Helicobacter genus	0/5
	M. pulmonis PCR	0/5
	P. pneumotropica-Heyl PCR	0/5
	P. pneumotropica-Jawetz PCR	0/5
	Salmonella genus PCR	0/5
	S. moniliformis PCR	0/5
	S. pneumoniae PCR	0/5
	Cryptosporidium PCR	0/5
	Entamoeba PCR	0/5
	Giardia PCR	0/5
	Mite PCR	0/5
	Pinworm PCR	0/5
	Spironucleus muris PCR	0/5

Table S2 - ***Analyses on MCB blood samples***

	Microorganism	Positive/Total
Human Comprehensive CLEAR Panel (Charles River)	Adeno-associated virus	0/2
	LCMV PCR	0/2
	Human cytomegalovirus	0/2
	HANT (Hantavirus Hantaan) PCR	0/2
	SEO (Hantavirus) PCR	0/2
	Hepatitis B virus	0/2
	Hepatitis C virus	0/2
	Epstein-Barr Virus	0/2
	Herpesvirus type 6	0/2
	Herpesvirus type 7	0/2
	Herpesvirus type 8	0/2
	Mycoplasma Genus PCR	0/2
	HPV-16	0/2
	HPV-18	0/2
	Parvovirus B19	0/2
	Hepatitis A virus	0/2
	John Cunningham virus	0/2
	BK virus	0/2
	Human Foamy Virus	0/2
	Human T-lymphotropic virus	0/2
	HIV-1	0/2
	HIV-2	0/2

	Criteria	Result
In vitro Assay for Detection of Adventitious Viruses Vero, MRC-5, NIH 3T3 and BHK-21 cell lines (Charles River)	Appearance of cytopathic effects (CPE)	None detected
	Appearance of hemadsorption using chicken, guinea pig and human erythrocytes	None detected
	Appearance of hemagglutination using chicken, guinea pig and human erythrocytes	None detected
	Appearance of inhibitory effects (interference control)	None detected
	System suitability criteria for a valid test	Fulfilled

Table S3 – Primers and TaqMAN probe sequences for high-sensitivity quantitative RT-PCR detection and quantification of *Pb*VAC.

Primer/ Probe	Sequence (5' → 3')
**Forward**	AGCATTAAATAAAGCGAATACATCCTTAC
**Reverse**	GGAGATTGGTTTTGACGTTTATGTG
**Probe**[1]	FAM TTC TTG CGT TTA CGA CAT G MGB

[1] – Probe labelled with 6-carboxy fluorescein (FAM) and with minor grove binding non-fluorescent quencher (MGB)

Uncropped pictures of agarose gels presented in the various figures of the manuscript

Fig. 3. *Pb*Vac parasite detection and quantification by high-sensitivity qRT-PCR.

A) Serial dilutions of a plasmid containing 18S ribosomal gene

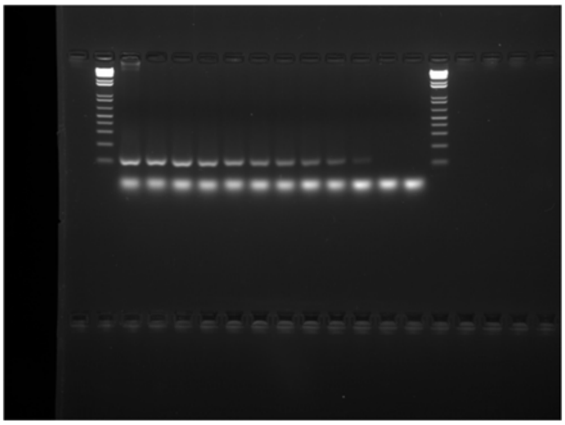

B) Serial dilutions of the PbVac parasite in mouse blood

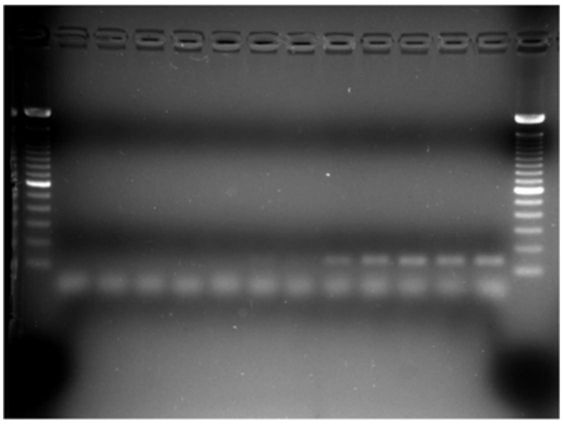

C) Serial dilutions of the PbVac parasite in human blood

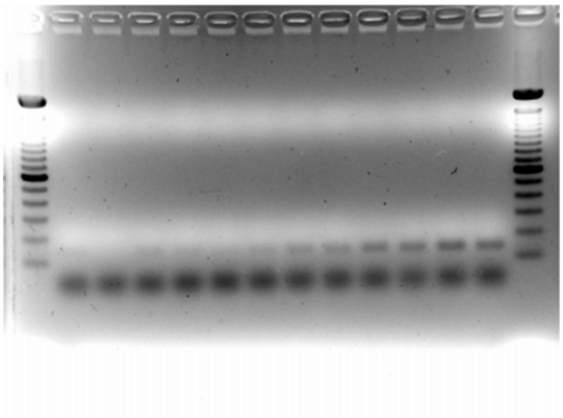

Fig. 4. ***Pb*****Vac tissue biodistribution in NZW rabbits.**

B) Gel electrophoresis analysis of the PbVac 18S ribosomal gene in various organs of male rabbits at different time points after parasite administration

1hpi

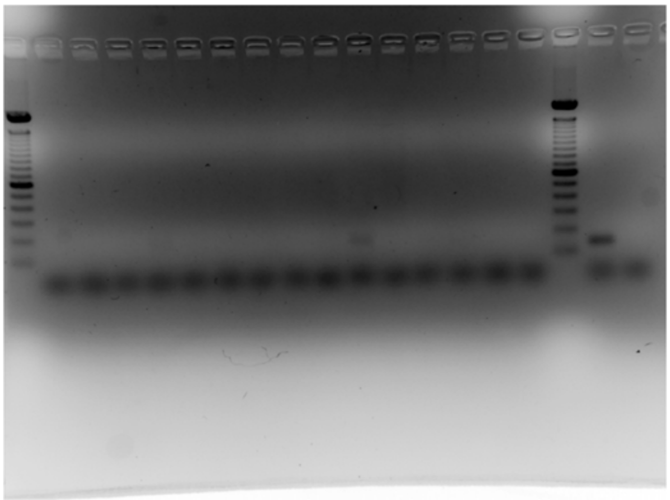

48hpi

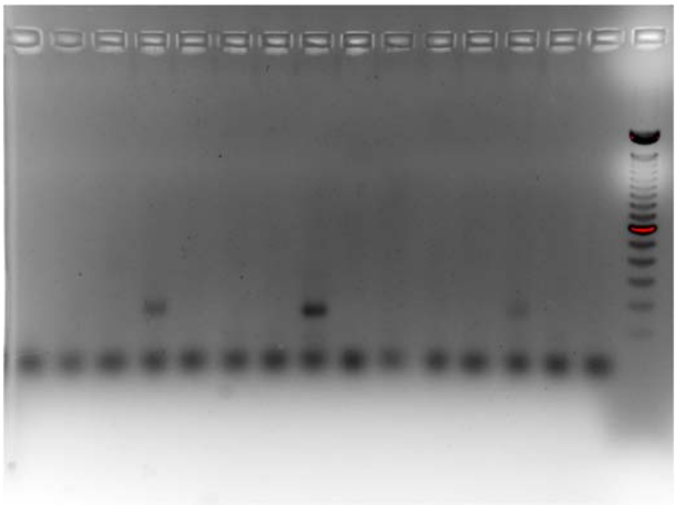

10dpi

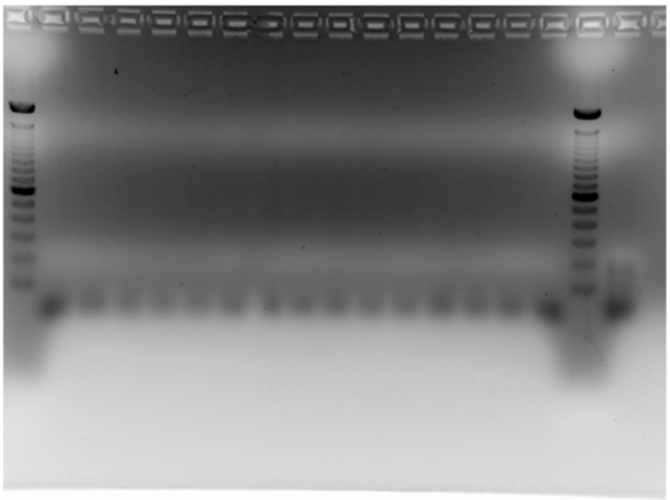

C) Gel electrophoresis analysis of the PbVac 18S ribosomal gene in various organs of female rabbits at different time points after parasite administration

1hpi

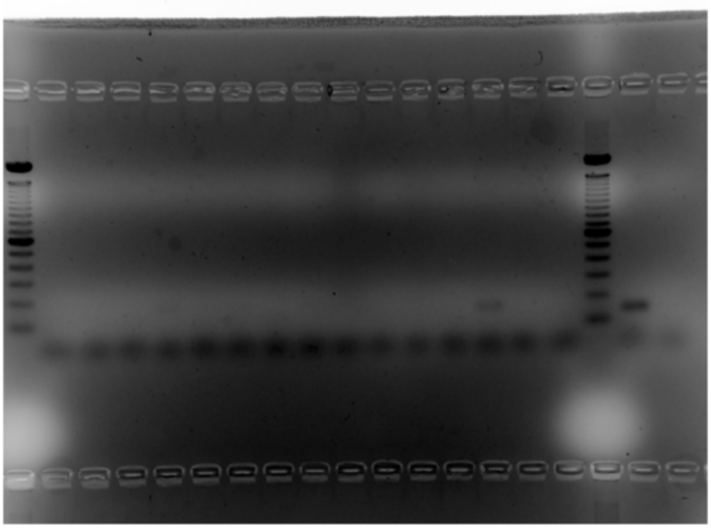

48hpi

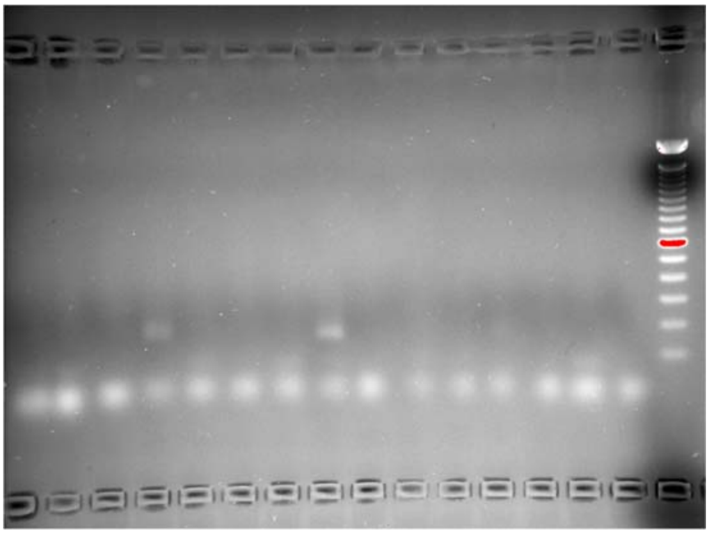

10dpi

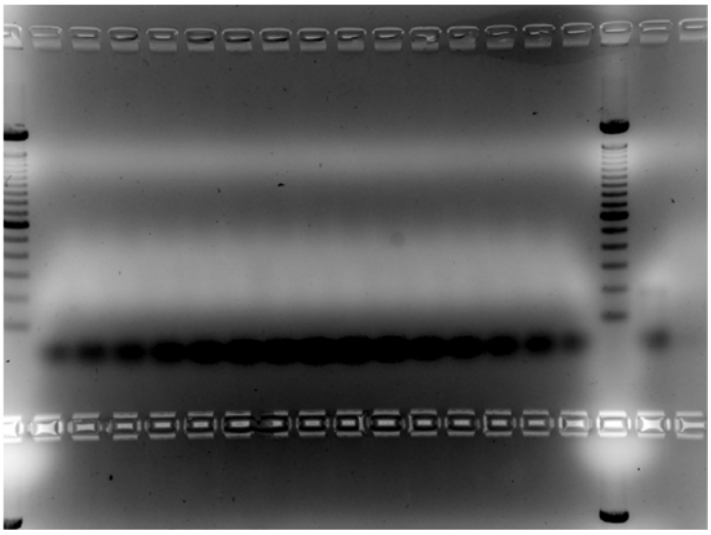

Fig S2. Representative gel electrophoresis analyses of the quality of RNA samples extracted from selected male and female rabbit organs at different times after parasite administration.

Gel 1

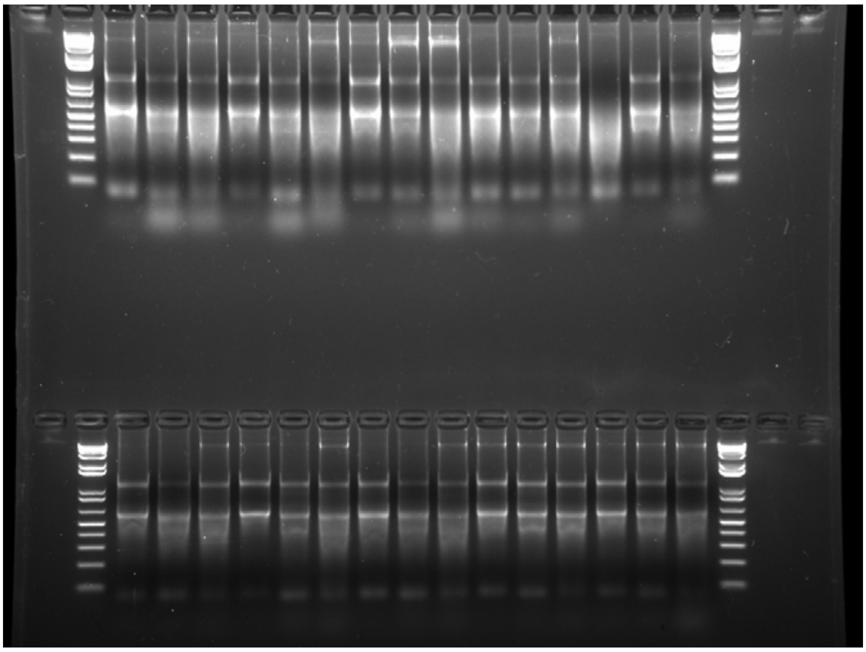

Gel 2

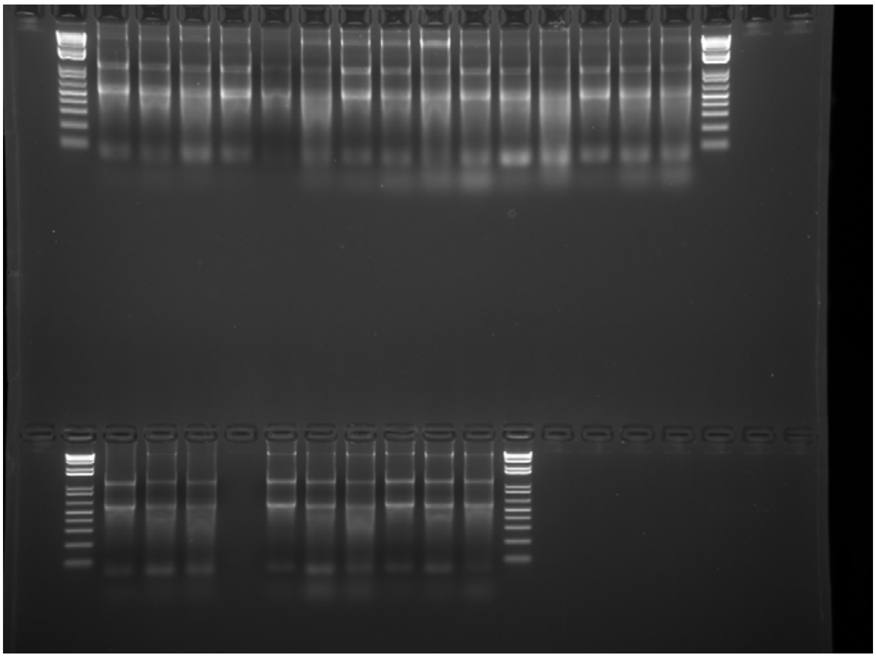

Fig S3. Detection of *Pb*Vac 18S ribosomal RNA extracted in the presence of selected "spiked" rabbit tissues.

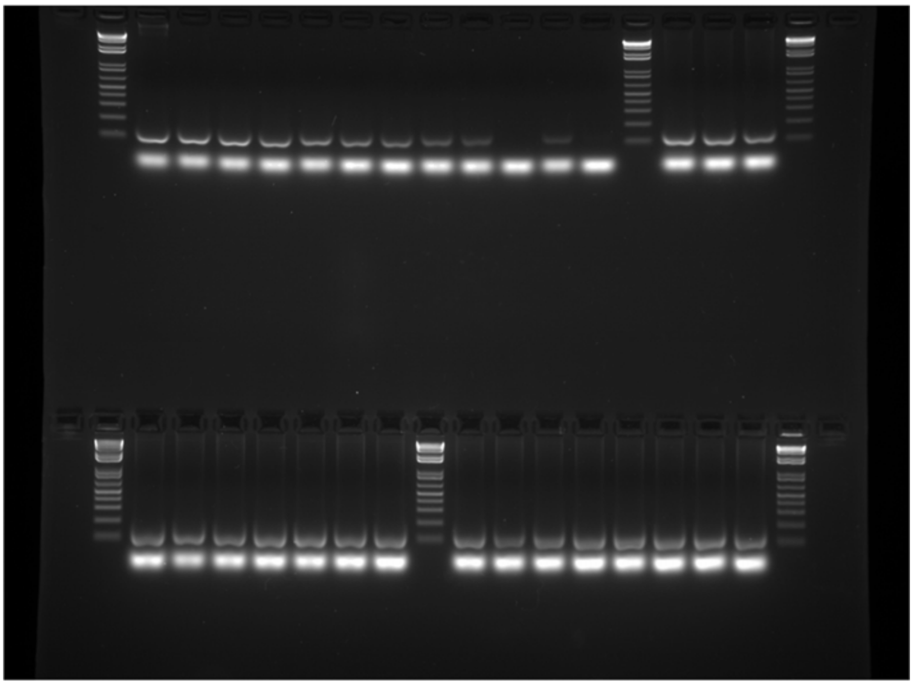

Supplement: Supplementary file 1 — Supplemental Material [file 41541_2018_91_MOESM1_ESM.pdf]
